# Relationship-building around a policy decision-support tool for urban health

**DOI:** 10.5334/bc.110

**Published:** 2021-08-23

**Authors:** Clément Deloly, Anne Roué-Le Gall, Gemma Moore, Lucy Bretelle, James Milner, Nahid Mohajeri, David Osrin, Giuseppe Salvia, Phil Symonds, Ioanna Tsoulou, Nici Zimmermann, Paul Wilkinson, Michael Davies

**Affiliations:** Department of Environmental and Occupational Health, School of Public Health (EHESP), Rennes, France; Department of Environmental and Occupational Health, School of Public Health (EHESP), Rennes, France; UMR CNRS Arènes, Université de Rennes, Rennes, France; Institute for Environmental Design and Engineering, Bartlett School of Environment, Energy and Resources, University College London, London, UK; Buro Happold, London, UK; Centre on Climate Change and Planetary Health, Department of Social and Environmental Health Research, London School of Hygiene and Tropical Medicine (LSHTM), London, UK; Institute for Environmental Design and Engineering, Bartlett School of Environment, Energy & Resources, University College London, London, UK; Institute for Global Health, University College London, London, UK; Institute for Environmental Design and Engineering, Bartlett School of Environment, Energy and Resources, University College London, London, UK; Institute for Environmental Design and Engineering, Bartlett School of Environment, Energy and Resources, University College London, London, UK; Institute for Environmental Design and Engineering, Bartlett School of Environment, Energy and Resources, University College London, London, UK; Institute for Environmental Design and Engineering, Bartlett School of Environment, Energy and Resources, University College London, London, UK; Centre on Climate Change and Planetary Health & Department of Public Health, Environments & Society, London School of Hygiene & Tropical Medicine (LSHTM), London, UK; Institute for Environmental Design and Engineering, Bartlett School of Environment, Energy and Resources, University College London, London, UK

**Keywords:** cities, evidence-based decisions, integrated knowledge translation, knowledge transfer, public health, public policy, research–policy engagement, science–policy interface, trust

## Abstract

Contemporary challenges linked to public health and climate change demand more effective decision-making and urban planning practices, in particular by taking greater account of evidence. In order to do this, trust-building relationships between scientists and urban practitioners through collaborative research programmes is required. Based on a policy-relevant research project, Complex Urban Systems for Sustainability and Health (CUSSH), this project aims to support the transformation of cities to meet environmental imperatives and to improve health with a quantitative health impact assessment. A case study in Rennes, France, focuses on the role of a policy decision-support tool in the production and use of knowledge to support evidence-informed decision-making. Although the primary objective of informing decision-making through evidence-based science is not fulfilled, the use of a decision-making support tool can lay the foundations for relationship-building. It can serve as a support for boundary-spanning activities, which are recognised for their effectiveness in linking science to action. This case study illustrates that the path of knowledge transfer from science to policy can be challenging, and the usefulness of using models may not be where it was thought to have been.

## Glossary of Organisations And Acronyms

CRAFT: Cities Rapid Assessment Framework for Transformation
CUSSH: Complex Urban System for Sustainability and Health
EHESP: Ecole des Hautes Etudes en Santé Publique (National School of Public Health)
GHG: greenhouse gas
HIA: health impact assessment
IKT: integrated knowledge translation
PCAET: Plan Climat-Air-Energie Territorial (Metropolitan Plan for Fighting Climate Change)
RBUS: Réseau Bretagne Urbanisme et Santé (Britany Healthy Urban Planning Network)
SPIs: science–policy interfaces

## 1 Introduction

Cities around the world face the formidable challenge of how to develop in ways that meet the increasingly urgent imperatives of planetary and population health (Singh & Beagley 2017). Action to date has been too slow to meet the ambitions of the 2015 Paris Agreement on climate change (UNFCC 2015) and most cities have unmet needs for environmental improvement for the health and wellbeing of their residents (Rogelj *et al*. 2016).

New, creative and experimental approaches are needed to accelerate change (Grant *et al*. 2017), including innovations in how researchers, policymakers and decision-makers work together. The use of scientific evidence and participatory engagement is widely recognised as important to the complex policy processes of environmental public health (Brundtland 1997; Cornell *et al*. 2013; United Nations 2019). However, the ‘producers’ of scientific evidence are usually separated from its ‘users’ (Cairney & Oliver 2017). There is an emerging body of strategies to broker relationships, communicate evidence or co-produce knowledge. For example, innovative ways to influence policymakers include tailor-made and trust-based research–practice partnerships (Farrell *et al*. 2019; Jagosh *et al*. 2015). This practice has been called ‘boundary spanning’: ‘work to enable exchange between the production and use of knowledge to support evidence-informed decision-making in a specific context’ (Bednarek *et al*. 2018: 1176). Boundary activities ‘can sustain productive interactions between science, policy and society, lead to increasingly useful science, and ultimately build capacity for science to inform decision-making about sustainability’ (Bednarek *et al*. 2018: 1177). More than a simple process of disseminating science (*e.g*. via communication, applied science or advocacy), the activities require sufficient time, resources and expertise. Specific pieces of ‘work’ on which different communities of practice are able to meet, exchange knowledge and initiate joint activities are known as ‘boundary objects’ (Star & Griesemer 1989) because they sit at the boundary between two social worlds (such as those of policymakers and scientists) and allow communication and coordination between them (Trompette & Vinck 2009). A range of approaches bring together policymakers and researchers, some of which lean to one-way linear knowledge-transfer models and others two-way models. Within this paper, boundary spanning refers to activities that aim to sustain productive (multidirectional) interactions between stakeholders.

The challenges of boundary spanning, or knowledge production and transfer, between research and policy are multiple and well documented (Bednarek *et al*. 2018; Cvitanovic *et al*. 2016; Fazli *et al*. 2017; Kothari *et al*. 2017; Naustdalslid 2011; Oliver *et al*. 2014; Taylor & Hurley 2016).

Collaboration and relationship-building between policymakers and researchers, including building trust and mutual respect, are among the key factors influencing knowledge production (Cvitanovic *et al*. 2016; Fazli *et al*. 2017) and the effective use of evidence by policymakers (Oliver *et al*. 2014).

## 2 Understanding and Evaluating Research-Policy Engagement

There is increasing recognition of the complex and contested nature of policy development and a growing body of research explores its use (or lack of use) of scientific evidence (Oliver *et al*. 2014; Weiss 1993). Current research questions the view that scientific evidence translates into policymaking through rational and linear processes, and acknowledges the roles of systems, relationships and different forms of knowledge at the interface between science and policy (Best & Holmes 2010; Edwards 2021; Pineo *et al*. 2020). Examining engagement practice at the boundary may help one to understand the dynamics between research and policymaking (Oliver *et al*. 2021). Without more information about the processes and effects of different approaches, it is likely that engagement activities will have limited impact on the goal of increasing the use of evidence in policymaking. In a recent systematic review, Oliver *et al*. (2021) identified a total of 1922 individual research–policy engagement activities (in 428 organisations) globally. Publicly available evaluations were available for only 3% of initiatives.

Despite the paucity of publicly available evaluations, there is a growing body of literature on how to effectively evaluate engaged research and the use of science in decision-making. For example, Edwards & Meagher (2020) present a framework to evaluate the impact of research on policy and practice, piloted in case studies led by a UK government research agency. The framework challenged linear, output-focused measures of ‘instrumental’ impact, attempting instead to understand the ‘building blocks’ to impact via three questions: What changed, why and so what? Used as a tool for self-reflection, this encouraged researchers to consider the relationships between processes that led, or could lead, to a richer understanding of impact in its broadest sense. In the field of environmental policy, Owens (2005) notes that much of the conventional wisdom about ‘policy-relevant research’ is grounded in rational and instrumental conceptions of the role of evidence and knowledge. Owens urges researchers to consider the ‘cognitive perspectives’ on the policy process in which research is deemed to have long-term, subtle, but nevertheless significant, effects. Evaluation may find a focus on the development of relationships or partnerships (Kothari *et al*. 2011), productive exchanges (Muhonen *et al*. 2019; Spaapen & van Drooge 2011), ideas and conceptual shifts, or the ‘enlightenment function’ of research on policy (Weiss 1977). The diverse levels and forms of evaluation indicate that there is no one-size-fits-all approach to evaluating engaged research and the use of science in decision-making; instead, the literature reveals that effective evaluation is designed, implemented and shared in relation to its context and purpose.

In the development and implementation of a coastal planning tool in the US state of Louisiana, Wong-Parodi *et al*. (2020) revealed some key insights to support meaningful integration of relevant science into the policy realms. The authors suggest that six characteristics should be taken into account in the development of decision support tools to enhance stakeholders’ ability to make ‘high-quality’ or evidence-based decisions: (1) to define the decision that needs to be made; (2) to identify alternatives; (3) to obtain relevant information and evidence; (4) to articulate values linked to the decision; (5) to evaluate alternatives; and (6) to monitor outcomes. Stakeholder engagement is a critical part of the process, and ‘user-generated input and feedback from tool utilisation helps guide improved DST use and usability’ (Wong-Parodi *et al*. 2020: 57). This echoes the work of Sarkki *et al*. (2015), who outline four key attributes for effective science-policy interfaces (SPIs): credibility, relevance, legitimacy, three attributes of knowledge production and exchange and iterativity, an additional attribute to better consider interactions and participants’ and external audiences’ perceptions of an SPI.

This paper explores the idea of decision support tools as boundary objects. Specifically, a case study is described and assessed involving the development and use of a Complex Urban Systems for Sustainability and Health (CUSSH) policy decision-support tool for urban health—the Cities Rapid Assessment Framework for Transformation (CRAFT)—with the French city of Rennes. A key question is the role of the CRAFT tool in developing and strengthening the collaboration and co-production of knowledge by researchers and city planning practitioners. Based on this experience, this study reflects on the exchange between the production and use of knowledge to support evidence-informed decision-making in Rennes.

## 3 Methods

An ethnographic approach is used to understand the complexity of how people think, feel, act and interact.^1^ Rather than adopting a traditional ethnographic approach, which tends to focus on a specific location or organisation, the multi-sited approach used here addressed the fact that CUSSH is a transdisciplinary, multi-agency and multi-context international programme with multiple networks and locations. Multi-sited ethnography (Marcus 1995) tends not only to ‘follow’ a range of evolving networks but also to build upon understandings of how and under what conditions strategic collaborations between researchers and participants can emerge from the fieldwork. This aligned well with the goals of CUSSH. Marcus (2011: 28) states that:

in some inquiries, fieldwork is not simply a schedule of interviews but is very often stage managing in collaboration with connected events of dialogue and independent inquiries around them.

There are a wide range of guidelines and tools for addressing such an open remit, including ‘following’ a certain object, idea or process. This study considers the development and use by different stakeholders and organisations of the CRAFT decision-support tool (as a boundary object). Although the wider evaluation of CUSSH uses a range of methods (including interviews, surveys and observations), the case study focused on the analysis of field notes and documents by two of the authors (C.D. and A.R.L.G.) to map the emergence and evolution of the collaboration between researchers and policymakers. This ethnographic approach, focusing on the CRAFT tools development and usage, enabled the authors to tap into the intricacies of the relationship between the stakeholders involved in the Rennes collaboration.

To understand the role of the CRAFT tool in the policy engaged research processes, a descriptive and chronological narrative was developed illustrating the interactions between CUSSH researchers and city stakeholders from 2016 to 2020. The first step in the building of this narrative was to identify, from records of meetings and email exchanges, the key CUSSH events (such as workshop, meetings, specific task, *etc*.) since the launch of the project. Using meeting notes, observation reflections and shared documents, 18 key CUSSH events were identified and then analysed to extract key themes (see Appendix 1 in the supplemental data online) and then mapped to programme theory. This work was carried out jointly by the two authors mentioned above, who were fully involved in the majority of these events. In dedicated working sessions, they examined and reflected together on the data collected and mapped out what happened. The narrative is summarised in a timeline in Appendix 2 in the supplemental data online.

In the next section, the stakeholders involved in the case study are outlined, a short background to the CRAFT tool is provided and an overview of the CUSSH programme theory used as a framework for reflection is given.

### 3.1 Project and Stakeholders

The Complex Urban Systems for Sustainability and Health (CUSSH) project aims to conduct policy-relevant, actionable research to support the transformation of cities to meet environmental imperatives, and to improve the health and wellbeing of current and future populations (Davies *et al*. 2021). Key questions are whether and how the use of scientific evidence, systems thinking, and participatory engagement in decision processes can strengthen the planning and implementation of environmental and health policies. The project involves a consortium of research partners in six cities across three continents. The project is transdisciplinary in aspiration and brings together groups of researchers, decision-makers (policymakers, health and urban planning professionals) and public groups in the development and use of research evidence.

CUSSH is a four-year research collaboration that aims to improve capacity to guide transformational health and environmental changes in cities. The programme seeks to promote city transformation for improved environmental quality, sustainability and health by bringing together groups of researchers, decision-makers such as policymakers, public health and built environment professionals, and public groups in the development and use of research evidence (Moore *et al*. 2021). The case study research was carried out in partnership with policymakers in Rennes, the regional capital of Brittany. Rennes has a long-standing interest in health issues and was the first French city to be selected by the World Health Organization’s (WHO) Healthy Cities project (Le Goff & Séchet 2011). In 2011, a local collaborative network was set up, the Réseau Bretagne Urbanisme et Santé (RBUS), involving public health researchers at the Ecole des Hautes Etudes en Santé Publique (EHESP) based in Rennes, and local decision-makers working on healthy urban planning for the city. This network, and more specifically the EHESP scientific team, supported and accompanied the work of implementing the CRAFT tool in Rennes and conducted this study. Public policy can be defined as:

the deliberate decisions—actions and nonactions—of a government or an equivalent authority toward specific objectives.

(Weible 2018: 2)

Of the many actors involved in setting public policy agendas, those affiliated with government taking policy decisions are considered policymakers and this is the definition being used within the wider CUSSH programme. The stakeholders involved in the case study are described in Table 1

### 3.2 Craft Tool

**Figure 1:**
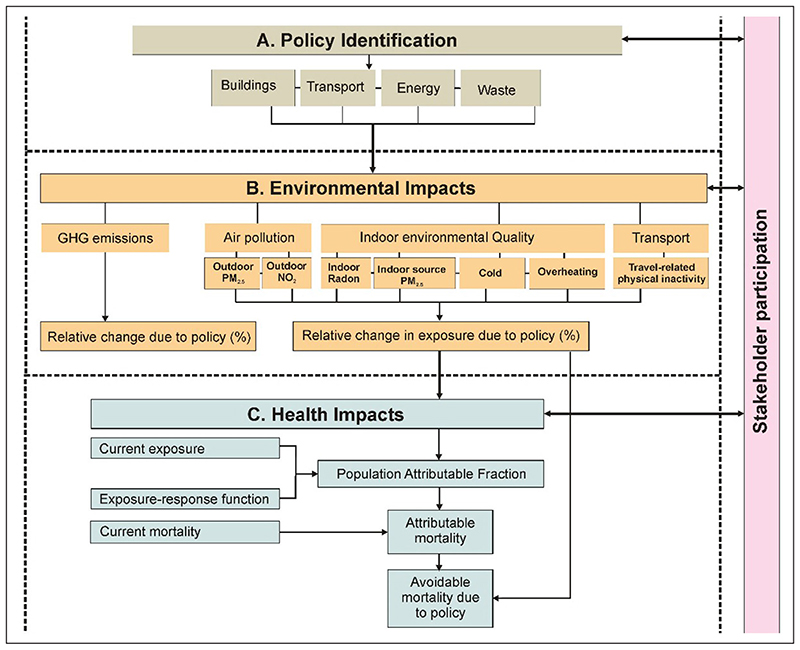
Flow diagram for the Cities Rapid Assessment Framework for Transformation (CRAFT) tool. *Note:* Beige indicates the first step of the assessment using the CRAFT tool: policy identification. Orange indicates the second step: environmental impacts. These include greenhouse gas (GHG) emissions, air pollution and indoor air quality, as well as travel-related physical inactivity. Blue indicates the final step of the assessment: health impacts. The arrows between stakeholder participation and the assessment steps represent engagement (*e.g*. presenting policy choices or showing model results) and receiving feedback from stakeholders. *Source:* Adapted from Symonds *et al*. (2021).

CRAFT is a quantitative health impact assessment (HIA) tool developed to allow rapid comparison of city policies in terms of their impact on environmental exposures, population health and greenhouse gas (GHG) emissions. First implemented in London, UK (Symonds *et al*. 2021), it is aimed at helping decision-makers understand the scale of the climate actions needed to meet policy ambitions and to show the potential health benefits or disbenefits of alternative policies. It is intended to provide a first rapid assessment of policy options and would often be followed by more detailed assessments and modelling once options have been narrowed (Figure 1).

In Rennes, it was applied to assess the impact of 10 selected objectives for the city relating to building refurbishment, transport, energy and waste, from the city’s Plan Climat-Air-Energie Territorial (PCAET) (Rennes Métropole 2019–24) approved by the administration of Rennes Metropole in April 2019. The aim of the PCAET is to achieve a 40% reduction in the city’s GHG emissions by 2030. The CRAFT tool provided a quantitative assessment of the impact of PCAET policies on GHG emissions as well as on exposures to environmental hazards to health (including air pollution), travel-related physical activity and associated mortality. Its results were used to inform decision-making processes and help prioritise policy options in discussions between the CUSSH research team and city policymakers. The results of the CRAFT analysis are not discussed in this paper. Instead, the focus is on the tool’s role in the relationship between scientists and city practitioners.

### 3.3 Cussh Programme Theory

**Figure 2:**
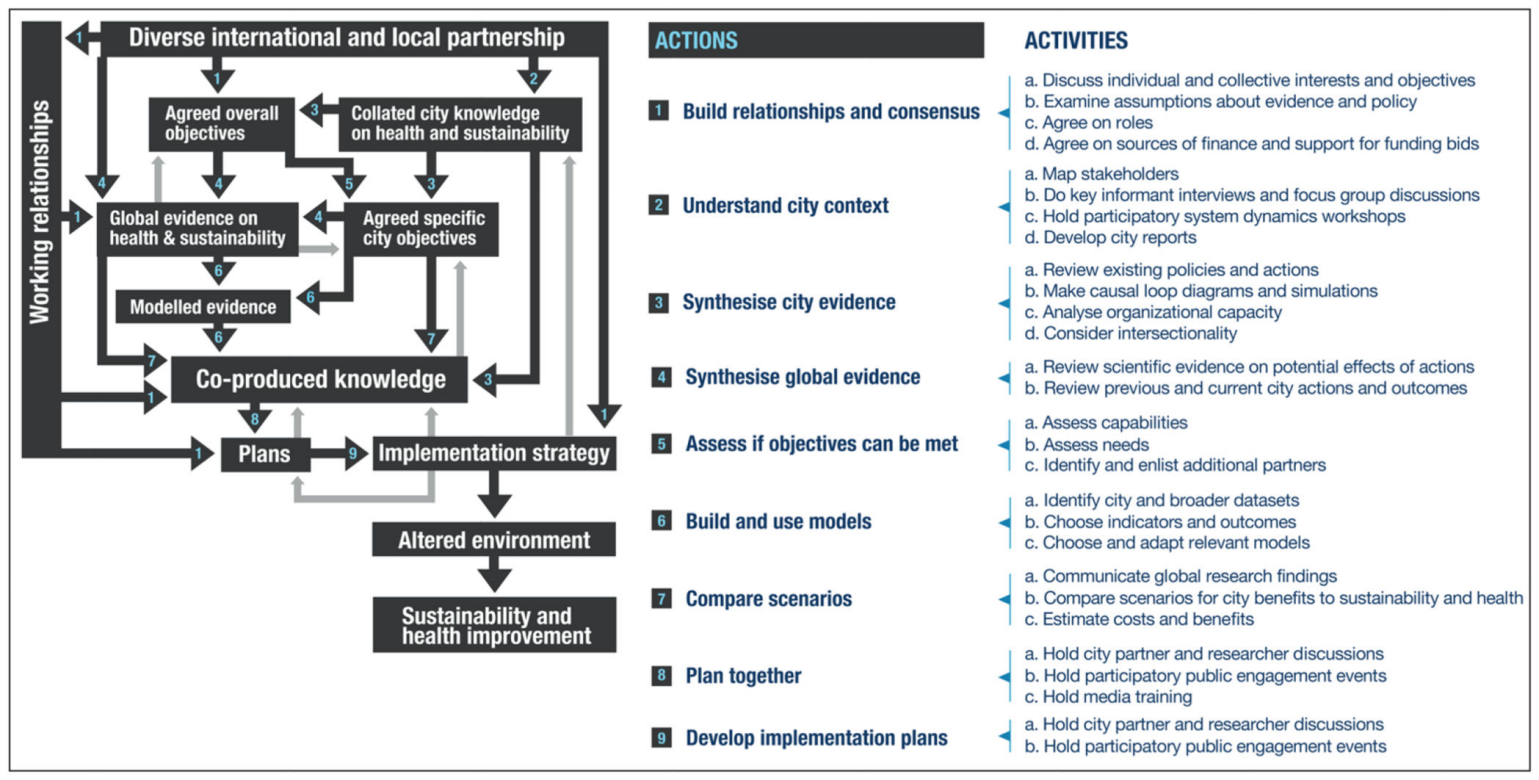
Complex Urban System for Sustainability and Health (CUSSH) action model. *Source:* Moore *et al*. (2021).

The CUSSH project has developed a programme theory to unpack and explain how collaborative engaged research will ‘work’ to achieve its desired effects (Moore *et al*. 2021). The programme theory was developed through a participatory process among the wider CUSSH consortium to ensure the input of a broad range of perspectives and create a shared understanding of the goals of the programme among team members. The programme theory comprises an ‘action model’ and a ‘change model’. The action model outlines how, in theory, the programme will help cities to achieve health and sustainability (Figure 2). Co-produced knowledge is a centrally important output and it is envisaged that the outlined stages culminate in co-produced knowledge and an implementation strategy for polices. There are 10 key stages (detailed in the results section), some of which mirror the suggestions of Wong-Parodi *et al*. (2020) for the design and development of evidence-based policy decision-support tools. The stages and processes are likely to be non-linear and iterative. The change model documents the expected changes from the programme, and what has led to them. It emphasises the ways in which the programme might affect people, organisations, collectives, research and practice (taking a broad view of impact as urged by authors including Edwards & Meagher 2020). The programme theory and its development are detailed by Moore *et al*. (2021).

Programme theories are provisional (Birckmayer & Weiss 2000), and evaluation and reflection play a key role in scrutinising what actually happens in practice as opposed to what is documented in theory. Within this case study, the CUSSH programme theory provide the foundations for the assessment of the CRAFT policy decision-support tool.

A CUSSH evaluation framework (in development, led by authors G.M. and D.O.) is tied to the programme theory. It is broken down into three parts: (1) output markers linked to an action model; (2) outcome markers linked to a change model; and (3) reflective questions and guiding principles. The reflective questions are tied to principles for complex, transdisciplinary, partnership projects and are adapted from scholars who have provided guidance for evaluation of such projects, predominately: Pineo *et al*.’s (2020) transdisciplinary model for health research; alongside Edwards & Meagher’s (2020) simplicity of ‘what changed, why, and so what?’; Davies *et al*.’s (2020) reflective questions for practitioners undertaking co-production in research; and guiding criteria for transformational research noted by Hölscher *et al*. (2021). Within this study, the CUSSH reflective questions have been used alongside the action model to provide an analytical framework to examine the role and value of CRAFT in enabling the co-production of knowledge and, ultimately, the use of evidence in policymaking.

## 4 Results: Understanding The Role of the Craft Tool

This section contains two parts. In the first subsection the case study is mapped against the CUSSH Programme Theory action model (Figure 2), to examine which research-policy engagement activities and mechanisms were adopted for the goal of improving evidence use in Rennes policymaking. The second subsection draws on researcher team reflections on how theses interactions have affected the collaboration.

### 4.1 Mapping the Rennes Case Study Against Programme Theory

The interactions within the case study were mapped against the action model, and are summarised in Table 2. The project started at the end of 2016 with provisional funding from the Wellcome Trust. Working relationships within the Rennes case study (*Action model step 1: Build relationships and consensus*) began in 2017 following a meeting between the CUSSH management team and the Deputy Mayor for Health of the city of Rennes (a Rennes stakeholder). An in-principle agreement was reached for joint work in Rennes and political support was obtained. This was followed by engagement and participation of other Rennes stakeholders, including city practitioners, decision-makers and actors engaged in local environmental, health and urban planning. The Deputy Mayor for Health shared the CUSSH project and its ambitions with various Rennes stakeholders, leading to a meeting between the CUSSH team and the RBUS network in May 2017.

Understanding the city context, *Action model step 2: Understand the city context* was initiated in June 2017 via a ‘kick-off workshop’ in Rennes; this workshop provided an opportunity for the CUSSH team to familiarise themselves with the geography of the area and to meet Rennes stakeholders such as the local scientific team, the EHESP team and other community practitioners working on health, environment and urban planning. It also allowed CUSSH researchers to present the aims of the programme and how the research team might work with local stakeholders. In addition to enabling CUSSH researchers to gain a better understanding of the local context (the different stakeholders, how they work together, the RBUS network and the different public policies), the meeting helped refine the project plan with a view to finalising contract responses to the Wellcome Trust. Initial discussions were held about possible forms of modelling as a decision-support tool for urban health.

In February 2018, the Deputy Mayor for Health took part in a workshop held in London with the aim of planning the CUSSH project and its governance, and to set out stakeholder roles and relationships and working relationships between the different cities. There was a return to *Action model step 1: Build relationships and consensus*, as the Deputy Mayor for Health continued their efforts to involve other Rennes stakeholders, including to confirm the input of EHESP which had extensive expertise on urban planning and health. This marked the beginning of direct liaison between the CUSSH scientific team and EHESP, a collaboration that will be formalised by the signature of an agreement at the end of October 2019.

*Action model step 3: Synthesise city evidence* commenced in May 2018, following a workshop in Rennes at which CUSSH scientific team members met Rennes stakeholders to retrieve local data for input into the CRAFT analysis. In May 2018, the PCAET was still being developed and the CRAFT model was understood by both stakeholder parties as a tool to support the implementation of the PCAET. Following the workshop, the CUSSH scientific team applied the CRAFT tool for the first time, paving the way for future exchanges between CUSSH researchers and Rennes stakeholders. These initial exchanges received widespread support from local actors as they served local political ambitions—leading to *Action model steps 4: Synthesise global evidence* and *5: Assess if objectives can be met* and continuing to feed *Action model steps 1–3.*

*Action model step 6: Build and use models*, had a number of sub-stages. In September 2018, the initial draft CRAFT results were sent electronically simultaneously to various Rennes stakeholders, including members of the RBUS network. The main objectives were to preserve the links with the local city stakeholders and to indirectly confirm their commitment to the research process. However, the reactions were rather negative and linked both to a lack of a clear explanation of the modelling calculation methods and a concern that the analysis would omit other important public policies. Despite the unfavourable reactions, the local scientific team at EHESP were keen to continue. In January 2019, they again met with Rennes stakeholders and concluded that there was interest in continued collaboration on the CUSSH project as an extension to the partnership that had been developed over several years by various Rennes city stakeholders, notably through the RBUS network. Exchanges with the research team then focused on improving the application of the CRAFT tool, which established a collaborative platform to exchange ideas and share information.

As clarifications about the project were needed, another meeting was held in January 2019 between the Rennes stakeholders and the deputy mayor, who again presented the project’s research objectives and potential benefits for the community of participation in CUSSH. This was a return to *Action model step 1: Build relationships and consensus*. At this point, the EHESP local scientific team and the Rennes stakeholders formally agreed to join the project, thus forming the CUSSH Rennes team as partner in the CUSSH project.

*Aligned to step 1 of the action model*, a scope of work was agreed in October 2019 (following a workshop with stakeholders held in September), which ensured that the collaboration met both the research requirements of the CUSSH project and the local ambitions of the city of Rennes. An agreed component was the CRAFT modelling, including its objectives, ways to communicate the results and validation. It was through the second iteration of CRAFT that the Rennes stakeholders officially joined the CUSSH project. Thus, within the case study *Action model steps 1* and *6* were intertwined.

The year 2020 was dedicated to implementing the agreed scope of work. In February, the CUSSH Rennes team travelled to London for a two-day workshop to discuss ambitions for Rennes. For the Rennes stakeholders, particular focus was put on making progress on the implementation of the agreed scope of work and the CRAFT tool. A half-day was devoted to CRAFT, which included discussion of the capacity of Rennes to take ownership of the CRAFT results and further understanding of the tool, its opportunities and challenges. It was decided that other local stakeholders should be involved in the process, particularly professionals responsible for the policies evaluated by the CRAFT process. The professional leading the PCAET was to be brought back into the discussion.

During April 2020 there was a return to *Action model step 5: Assess if objectives can be met*, as Rennes stakeholders requested the evaluation of new public policies with CRAFT, particularly policies related to mobility and urban development in the Rennes area. The Rennes team provided a matrix classifying these new objectives for CRAFT. Many exchanges followed to agree the correct translation of the policies from French to English and the corresponding revision of the CRAFT objectives.

Between May and October 2020, the Rennes CUSSH team elaborated an explanation note on the CRAFT tool and its results, addressed to the Rennes stakeholders in charge of PCAET. The repeated discussions about the CRAFT tool with the Rennes CUSSH team in the previous months were crucial here as they enabled them to provide a detailed explanation to avoid misunderstanding or confusion.

On 27 October 2020, the Rennes CUSSH team shared updated CRAFT results with the PCAET practitioner. The aim of this meeting was to understand how CRAFT results could inform the PCAET and its implementation, particularly by prioritising actions with the greatest benefits for the environment and health. Although the practitioner found the health calculations interesting, as no such calculations had previously been used to develop the PCAET, they expressed scepticism about the approach, specifically with regard to (1) the input data used, (2) the scale of the analysis (which was deemed too wide, making the results impractical), (3) the lack of novel insights—indeed, the results confirmed what was already known, such as the fact that mobility objectives have co-benefits for health and building refurbishment may entail some negative consequences on health, and (4) the lack of evidence relating to wellbeing and quality of life.

Despite this critical reaction, the practitioner expressed an interest in continuing the analysis by updating some of the input data in order to obtain more robust modelling results. They pointed out that when the PCAET was instigated health had not been identified as a major decision-making criterion by the elected officials and city managers; neither had been the prioritisation of certain objectives. For this reason, the approach supported by the CRAFT tool was not a priority for the development of the policy in question.

The CUSSH team in Rennes have since contacted the practitioner in charge of the PCAET several times to receive improved data in order to update the CRAFT results accordingly and attempt to improve collaboration. However, to date and despite several reminders, data have not been shared. A forthcoming meeting with the new elected representative in charge of urban planning will perhaps help with this.

What is clear from this analysis is that all the stages of the action model have not yet been achieved. Furthermore, there was a repeated return to the *Action model step 1: Build relationships and consensus* throughout the collaboration. This step has been key within the case study, emphasising that setting up the research partnership was not a linear process, but that the implementation of CRAFT served as a fundamental tool to strengthen it.

The CRAFT tool had unexpected effects on communication and cooperation between researchers and stakeholders. It did not allow a rapid evaluation of public policies, as its results did take time to be generated and are not yet complete, and its results did not influence policy (*Action model steps 7–10*). Yet, it has contributed substantially to establishing a partnership between researchers in Rennes and London and local stakeholders, paving the way for future collaborations.

### 4.2 Reflections at the Boundary Between Research and Policy

The narrative suggests that CRAFT and its results did not materially influence public policy (*i*.*e*. the PCAET), but did aid the exchanges between the Rennes stakeholders, the local scientific team and the wider CUSSH team. Although not having met the objective of ‘evidence use in policy’, the tool played an important role in the establishment of a partnership.

Even if the results of the CRAFT tool were not transferred into policy, the work undertaken by the different actors involved in its implementation has made the boundary between scientists and city practitioners more porous. Indeed, the work relating to the implementation of the CRAFT tool (the *building and use of models step of the action model*) enabled the various actors to find out more about the other stakeholders involved in the project. The Rennes stakeholders have a better understanding of (1) the specificity of the CUSSH project (*i*.*e*. the systematic and simultaneous consideration of public health and environmental issues in public development policies), (2) how the wider community could benefit from the project (notably by taking advantage of public health and environmental assessment tools for public planning policies that are decision-making tools), and (3) what science had to learn from this collaboration (notably by ensuring that scientific research is tailored for consideration within decision-making processes). The CUSSH scientific team also have a better understanding of (1) how the Rennes stakeholders interacted with each other (functioning of the RBUS network, the area’s ambitions, interactions between different public policies) and (2) the willingness of the actors in the area to consider public policies in a more systemic and qualitative way. These elements have contributed to making the ‘boundary’ between the researchers and policymakers more porous and to making the research partnership between practitioners and researchers stronger.

**Figure 3:**
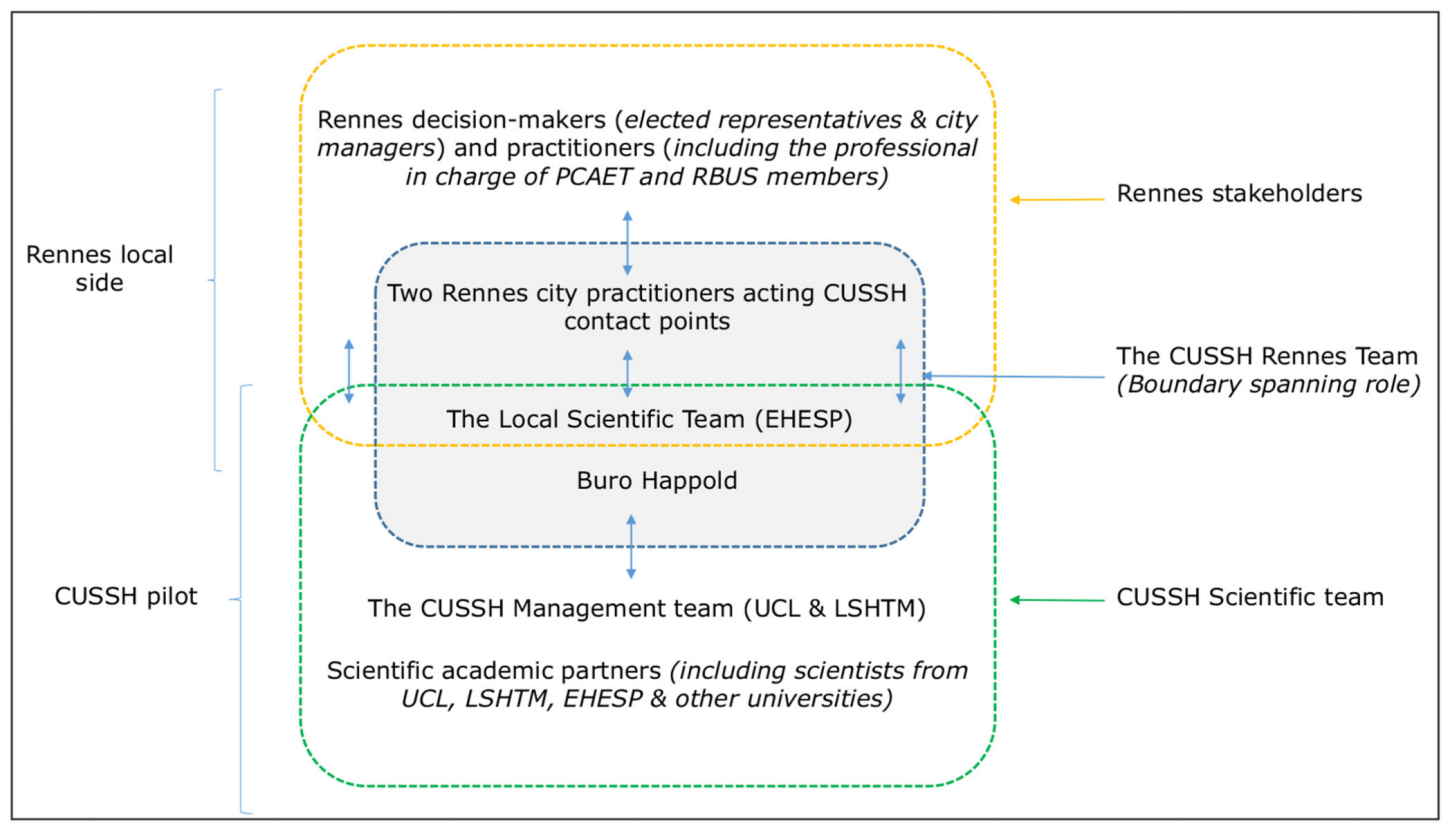
Mapping of stakeholders and governance organisation three years after the launch of the Complex Urban System for Sustainability and Health (CUSSH) project in Rennes.

It should be noted that the implementation of these different tasks was made possible by the local scientific team (EHESP), and a collaborator from an engineering consultancy bringing together city practitioners and the CUSSH scientific team. Figure 3 summarises the role of the local scientific team as boundary spanners (Bednarek *et al*. 2018; Gieryn 1983; Posner & Cvitanovic 2019):

an organisation that specifically and actively facilitates the process of exchange between the production and use of knowledge by dedicating their time to creating and enabling effective knowledge exchange.

## 5 Discussion

This case study has contributed insights into the production and use of knowledge to support evidence-informed policymaking. The present findings on the evolution of interactions between researchers and local stakeholders suggest that the CRAFT tool was catalytic in the implementation of the CUSSH project in Rennes, not for the results it produced (which were received with some scepticism and are not yet definitive), but for the exchanges it promoted, and that these exchanges were fundamental to building trust between the stakeholders. An understanding of the process of research and policy engagement was created by mapping it against an agreed programme theory. In doing so, this research has also highlighted the iterative cycles of actions that helped to strengthen relationships. In this section, the findings are considered in relation to existing research and theory. First, the strengths and limitations of the approach used are described. Then the role of tools, such as CRAFT, are discussed as representative functions in order to share the positions and identities of researchers and the research project. The findings are then linked to previously reported ingredients of successful science–policy collaborations and how the case relates to evaluative frameworks already in use. Finally, the importance of taking a relational perspective to practice is considered, with an emphasis on the role of building relationships in the exchange of knowledge.

This case study adopted a multi-sited ethnographic approach to explore and reflect upon the development of a policy decision-support tool. Its credibility is reinforced by the alignment of findings with the wider literature. However, there are limitations. Although this strategy is believed to be appropriate for this area of enquiry, the ethnographic approach focused on CUSSH team perspectives rather than on the views and experiences of other partners. The study was undertaken at the same time as the development of the CUSSH programme theory and supporting evaluation framework, and therefore was something of a testbed for the evaluation of the wider programme. Given the limitations, the analysis was based on reflections of two key researchers, whilst acknowledging that this is a starting point for wider evaluation and research.

This study has shown that, as a tool for modelling the environment and health impacts of public policies, CRAFT plays a role in bringing together different actors whose ambition is to promote policies that prioritise environment and health issues, building a shared goal or consensus. The CRAFT tool also had a *representative function* (Vinck 1999) in the project, *i*.*e*. it ‘represents’ those who conceived it (the CUSSH scientists), their intentions, perspectives, identities (to a degree) and therefore the CUSSH project itself. This representative function developed over the various workshops bringing together the CUSSH Rennes team and the CUSSH scientific team. Through discussions of the CRAFT tool, the CUSSH Rennes team gained a better understanding of what the CUSSH project was about. Reciprocally, the exchanges and reactions of the city stakeholders about CRAFT enabled the CUSSH scientific team to get to know them.

One of the primary objectives of the CRAFT tool was to inform policymakers about the potential impact of selected policies on environmental and health outcomes. Even though this may change over time with a new phase of modelling, this was unsuccessful. The reasons for the lack of success in this objective may be illuminated by what Sarkki *et al*. (2015) teach about the four ingredients of effective SPIs—and therefore for the effectiveness of the CUSSH research partnership: credibility, relevance, legitimacy and iteration.

During the presentations of the preliminary CRAFT results by the researchers, it was precisely the *credibility*, defined as:

the perceived quality, validity and adequacy and reliability of the knowledge, evidence and arguments exchanged at the interface

(Sarkki *et al*. 2015: 507)

of the results that was questioned.

Moreover, on both occasions the Rennes practitioner in charge of the PCAET told the CUSSH team that this sort of analysis was not a priority for the elected representatives, thus calling into question the *relevance*—defined as:

the ability to match knowledge with policy and societal needs, and the extent to which knowledge is usable

(Sarkki *et al*. 2015: 507)

of the approach.

Finally, the PCAET practitioner indicated that they did not have enough time to dedicate to their participation in the approach, thus limiting the *legitimacy*—which refers to:

the perceived fairness and balance of the science–policy interactions processes

(Sarkki *et al*. 2015: 507)

of the approach.

These three elements were not met. To gain credibility, the PCAET practitioner could have been more actively involved from the beginning of the CRAFT analysis and ‘their’ data should have been included from the outset. This was not possible as they said that they lacked time to dedicate to the CRAFT analysis, partly because health was not a key priority for the PCAET.

Second, as neither health nor the prioritisation of certain objectives had been identified as major decision-making criteria by the elected officials during the elaboration of PCAET, the approach supported by the CRAFT tool was not a priority for their department. The situation may have been different if the PCAET practitioner had been involved in the early stages of the development of the CRAFT tool; early involvement may have supported ownership, relevance and legitimacy (Jagosh *et al*. 2015). Convincing elected officials and management to include health in policymaking would have brought those three elements together and contributed to the effectiveness of the research partnership. This relates to what Jagosh *et al*. (2015) refer to as co-governance between actors in the research project, which often results in ripple effects from science-policy collaborations.

Although the first three ingredients (credibility, relevance and legitimacy) were not achieved, the process did entail *iteration* between researchers and stakeholders as:

a continuous multi-directional interaction that goes beyond simple repetition, building on previous practices, learning from success and failure, and fostering evolution of constructive relationships and knowledge itself among all participants at the interface, and between SPIs and external audiences.

(Sarkki *et al*. 2015: 507)

Indeed, the PCAET practitioner affirmed their interest in the approach and a willingness to pursue it, specifically by providing CUSSH with more robust data to improve credibility and legitimacy.

Finally, even if the CRAFT tool has brought the CUSSH Rennes team and the CUSSH scientific team closer together and has played a role in greater involvement of the Rennes stakeholders in the CUSSH project, it is clear that it has not yet led to evidence being used in policy formation. This means that the CRAFT tool cannot, strictly speaking, be considered a ‘boundary object’. It has certainly made it possible to put in place the necessary conditions for boundary-spanning activities (Bednarek *et al*. 2018), making the exchange of knowledge possible in future. Scrutinising the engagement process against the CUSSH action model showed that the step of *building of the model* overlapped the *building relationships and consensus* step. The present reflections highlight that the CRAFT tool played a significant role in establishing trusting relationships, initiating connections between the various participants, and involving decision-makers who may ‘use’ the information in future. By unpacking the processes of engagement, the findings illustrate the importance of taking a ‘relational’ perspective on the use of science in policymaking. Edwards (2021) points to the value and crucial role of the ‘relational’ aspects of collaboration between research and practice. Edwards introduces three key concepts drawn from a social–cultural perspective: relational expertise, common knowledge and relational agency. Relational expertise refers to the capacity to work relationally with others on complex problems. Edwards notes that this expertise is required to elicit and be explicit about what matters for each other’s professions. Relational expertise can result in the building of ‘common knowledge’, made up of the motives of the participating professionals. Her third concept, ‘relational agency’, refers to professions working together on joint action to solve a problem. Although the CRAFT tool has not led to agency or action, it has been key to developing relational expertise amongst team members, instigating discussions that have led to knowledge sharing among research and practitioners and the building of a partnership. These partnerships will be the platform on which to co-produce knowledge on health and sustainability solutions.

## 6 Conclusions

Although the Cities Rapid Assessment Framework for Transformation (CRAFT) tool has not yet fulfilled its primary objective (communicating evidence to inform the development of public policies), the interactions that revolved around its use made it possible to put in place the conditions for an effective research partnership. By making the boundary between stakeholders more porous and by helping to clarify individuals’ roles, the implementation of the CRAFT tool has resulted in the building of trust among stakeholders and ‘ripple effects’ (Jagosh *et al*. 2015) beneficial to Complex Urban Systems for Sustainability and Health (CUSSH) interactions: contacts with elected representatives, the beginning of modelling, discussions of policy priorities and understanding of different objectives.

The CRAFT policy decision-support tool did raise an awareness of health issues among decisionmakers and, above all, improved collaboration (Dannenberg 2016). This aligns with the second of the four categories of effectiveness of health impact assessments proposed by Wismar *et al*. (2007: 21): ‘awareness raising but no specific change is made to the decision’.

These results add to other studies on research–practice partnerships, whilst contributing to ongoing discussions on the impacts of such collaborations. The lessons learned from the authors’ experience of bringing together researchers, policymakers and practitioners at the city level with the intention of co-producing knowledge feeds into the integrated knowledge translation (IKT) community of expertise currently under development (Kothari *et al*. 2017). The present research also raises questions about how such engaged research programmes are evaluated and reflected upon (both within the programme and beyond), contributing to critical, reflective understandings of the ‘success’ of such partnerships.

## Supplementary Material

Supplemental data for this article can be accessed at: https://doi.org/10.5334/bc.110.s1

Supplementary Data

## Figures and Tables

**Table 1:** Overview of the different stakeholders grouped in six different teams.

TEAMS INVOLVED	STAKEHOLDERS INVOLVED
Rennes city practitioners acting as CUSSH contact points	Two members from the city of Rennes act as contact points for the CUSSH project (a project manager from the urban planning department and the head of the health-environment department of the city of Rennes
Rennes stakeholders	Rennes decision-makers (elected city representatives) and practitioners, including the Rennes city practitioners CUSSH contact points
Local scientific team	Researchers from EHESP
CUSSH Rennes team	Local scientific team (EHESP) in collaboration with the engineering consultancy Buro Happold and city practitioners CUSSH contact points
CUSSH management team	CUSSH project pilot team (UCL plus LSHTM)
CUSSH Scientific team	Academic researchers (including the local scientific team)

*Note:* CUSSH = Complex Urban Systems for Sustainability and Health; EHESP = Ecole des Hautes Etudes en Santé Publique (National School of Public Health); LSHTM = London School of Hygiene and Tropical Medicine; UCL = University College London.

**Table 2:** Rennes case study mapped against the Complex Urban Systems for Sustainability and Health (CUSSH) action model from the programme theory.

ACTION MODEL STEP	ACTIVITIES IN THE RENNES CASE STUDY	PERIOD
1. Build relationships and consensus	• Meeting between the CUSSH management team and the Rennes Deputy Mayor for Health → In-principle agreement reached	Early 2017
	• Deputy Mayor for Health shared the CUSSH project and its ambitions with other Rennes stakeholders and had a meeting between the CUSSH team and the RBUS network	May 2017
	• Rennes workshop dedicated to the CUSSH	May 2018
	• Involvement of Rennes stakeholders including EHESP in the CUSSH project	January 2019
	• CUSSH annual meeting—participation of a Rennes stakeholder	May 2019
	• Signed agreement formalising EHESP involvement in the CUSSH project	October 2019
	• Scope of work agreed following the September 2019 workshop with stakeholders	October 2019
	• Formal collaboration agreements signed between the EHESP and the CUSSH management team, and between the EHESP and Rennes stakeholders	October 2019–February 2020
2. Understand the city context	• ‘Kick-off workshop’ in Rennes in June 2017 → Project plan refined with a view to finalising contract responses to the Wellcome Trust	June 2017
	• CUSSH workshop in London with the Deputy Mayor for Health → Discussion of possible forms of modelling to support interactions between researchers	February 2018
3. Synthesise city evidence	• CRAFT modelling tool introduced: retrieve local data	May–September 2018
4. Synthesise global evidence	• Evidence-based synthesis and briefing reports produced	February–May 2018
5. Assess if objectives can be met	• Rennes stakeholders requested the evaluation of new public policies with the CRAFT tool, particularly public policies related to local mobility and urban development → Rennes team provided an updated matrix classifying new objectives for the CRAFT tool → Agreement on the correct translation of policies from French to English and corresponding revision of the CRAFT objectives	April 2020
6. Build and use models	• CUSSH scientific team applied the CRAFT tool o Draft CRAFT results sent electronically simultaneously to Rennes stakeholders, including members of the RBUS network o Exchanges with the research team focused on improving the application of the CRAFT tool o Two-day workshop to discuss CUSSH ambitions for Rennes with a particular focus on the CRAFT tool o Rennes CUSSH team shared the updated CRAFT results with an explanation note for the CRAFT tool with the city practitioner leading PCAET	May 2018–October 2020 September 2018 October 2018–Oct 2020 February 2020 October 2020
7. Compare scenarios	• Not yet achieved	
8. Plan together	• Not yet achieved	
9. Develop implementation plans	• Not yet achieved	
10. Implement plans	• Not yet achieved	

*Note:* CRAFT = Cities Rapid Assessment Framework for Transformation; EHESP = Ecole des Hautes Etudes en Santé Publique (National School of Public Health); PCAET = Plan Climat Air Energie Territorial (Metropolitan plan for fighting climate Change); RBUS = Réseau Bretagne Urbanisme et Santé (Britany Healthy Urban Planning Network)

## Practice Relevance

The requirements for an effective policy-relevant research programme depend on the establishment of trust. An analysis of the evolution of relationships between cities’ practitioners and scientists shows the need to establish relationships between stakeholders involved in this kind of research. Even if it does not directly support the implementation of transformational policies for health and sustainability, the use of a decision-making support tool can become essential in building trust relationships and later have ripple effects to achieve its primary goals. Moreover, a dedicated team has a key role to facilitate the links between scientists and cities practitioners—to boundary span. Finally, this paper adds to a growing body of work reflecting upon the success of policy–research partnerships.

## Footnotes

1
**Note**
The CUSSH project arose from the understanding that cities are complex systems characterised by diverse priorities, mutual interdependences, feedback relationships and inherent delays, making it difficult for decision-makers to anticipate the consequences of their actions (Davies *et al*. 2021). A systems thinking approach, *i.e*. an approach able to account for interdependencies, feedback and delays, is needed to understand and address this complexity (*e.g*. Richardson 2011).
