# Supplementary material for: Relationship-building around a policy decision-support tool for urban health
Source: Build Cities. Author manuscript; Available in PMC 2021 Oct 25. (PMC7611888; doi:10.5334/bc.110)
Supplement: Supplementary Data [file EMS137023-supplement-Supplementary_Data.zip › bc-110_roue-leGall/s1-bc-110_roue-leGall.pdf]

CUSSH Project phase	Stage	Dates	Event	Analysis
An interest in the CUSSH project from the Rennes political side	1	End of 2016	CUSSH Project pre-selected by Welcome Trust	**First necessary step** to set up CUSSH project
	2	May 2017	Health deputy mayor of the city of Rennes presents CUSSH to Rennes stakeholders	Because of the parallels between the CUSSH project and the priorities of the local health elected deputy mayor, the implementation of the CUSSH project in Rennes received **strong political support**.
	3	June 2017	Kick-off workshop in Rennes	The CUSSH management team learned more about **Rennes, its institutional landscape, 'territorie's' ambitions as reflected in public policies**.
	4	February 2018	Workshop in London to design the CUSSH Project with the health deputy mayor of the city of Rennes	After the official launch of the CUSSH Project, awarded by the Welcome Trust in December 2017, this workshop in London involved the health deputy mayor of the city of Rennes, demonstrating **political will** to get involved in CUSSH.
Negative exchanges on CRAFT, but nevertheless, interactions	5	May 2018	Workshop in Rennes for the CUSSH management team to meet the local scientific team and Rennes stakeholders	This workshop was an opportunity **to create contacts among the CUSSH management team, the local scientific team, and the Rennes stakeholders.** CUSSH work was launched to serve the implementation of local ambitions through CRAFT**, in support of the Rennes stakeholders.**
	6	September 2018	RBUS meeting to receive feedback on CRAFT's first round of results	Despite the shared good intentions of the CRAFT modelling, the results were not favourably received by the Rennes stakeholders, **because of the methodology used and because of the lack of understanding of local public policies**.
	7	December 2018	The local scientific team and Rennes stakeholders meeting about CRAFT	The initial local reaction on the first version of CRAFT could have led to a definitive end to any collaboration between the Rennes stakeholders and the CUSSH scientific team. However, **continuing exchanges led to further mutual understanding between the CUSSH scientific team and the Rennes stakeholders, encouraging ongoing collaboration**.
From political interest to the Rennes practitioners' interest	8	January 2019	The local scientific team and Rennes stakeholders agree to get involved in the project	In order to continue working together and avoid past mistakes, **the Rennes stakeholders needed to clarify the ambitions of the CUSSH project as well as the methods used in this project**. The health deputy mayor of the city of Rennes initiated this exchange, further demonstrating the local **political interest** of the CUSSH project has.<br>This stage marks the launch of the co-drafting of the 'scope of work'.

	9	May 2019	CUSSH Annual meeting	The participation of a Rennes stakeholder in this meeting proves that **the implementation of CUSSH is now supported by Rennes**, **due to a better understanding of the city practitioners' needs.**
	10	September 2019	Workshop at Rennes	This workshop was an **opportunity to discuss the CRAFT tool, providing a better understanding of its offer for decision-making support.** It was agreed that the implementation of CRAFT would be one of the first tasks to undertake. **The CRAFT tool thus represents an entry point into the CUSSH project for the Rennes stakeholders.**
	11	October 2019	Finalisation of the co-development of the scope of work, including the CRAFT tool	The **co-development** of the 'Scope of Work', including the CRAFT tool, between Rennes stakeholders, the local scientific team and the CUSSH management team was initiated in order **to provide a clear and precise framework for collaboration between Rennes and the CUSSH team**. This co-development re-established trust between these stakeholder.
	12	October 2019 – February 2020	Agreement signatures	**The collaboration and stakeholder roles were formalised in this stage.** Before then, stakeholders had found it difficult to understand their role in the implementation of the CUSSH project in Rennes.
Scope of Work implementation, including CRAFT among other tasks	13	From January 2020	The local scientific team takes part in the CUSSH meetings and its role is increasingly defined	The local implementation of the project becomes well established **through regular exchanges between the CUSSH management team and the local scientific team. Roles are now well-defined.** This stage marks the launch of a new local governance organisation.
	14	February 2020	London and Rennes workshop in London	This workshop allowed stakeholders from Rennes and London **to better understand the impacts of the CUSSH project on local ambitions**. Discussions around CRAFT allowed the Rennes stakeholders **to better understand the added value of this tool** and served to mobilise them in the implementation of this task.
	15	April 2020	Sending new objectives to the CUSSH team to be evaluated by CRAFT	Identifying new objectives to be evaluated was the opportunity **to initiate a close collaboration between the CUSSH Rennes team and the CUSSH scientific team**. It helped **the Rennes stakeholders to gain a greater understanding of the CRAFT tool, and enabled the CUSSH scientific team to better understand the needs of the Rennes stakeholders and comprehend public policies in a systemic way.**

	16	April 2020 to October 2020	Consolidation of the Rennes CUSSH team	**Co-editing a presentation note of the CRAFT tool and its results** to the person in charge of the PCAET and **setting-up local co-governance through bi-monthly meetings among the CUSSH Rennes Team to implement the Scope of Work** consolidated the CUSSH Rennes team.
	17	October 2020	Presentation of CRAFT results to a Rennes stakeholder	Despite a certain interest in the CRAFT tool by two Rennes practitioners serving as CUSSH contact people, the **knowledge produced by the CRAFT analysis was contested and the results did not succeed in influencing the implementation of the public policy (PCAET)**. Nevertheless, the lead of the PCAET has shown some interest in including health impacts in the evaluation of the PCAET.
	18	December 2020	Meeting between the CUSSH management team, the CUSSH Rennes team and the new health deputy mayor	Following the election of a new municipal team, local political support was crucial for the establishment of the CUSSH project locally. **Thus, the CUSSH management team and the CUSSH Rennes team met the new health deputy mayor of the city of Rennes**. This demonstrated the established trust between these two groups of actors.
