# Supplementary material for: Relationship-building around a policy decision-support tool for urban health
Source: Build Cities. Author manuscript; Available in PMC 2021 Oct 25. (PMC7611888; doi:10.5334/bc.110)
Supplement: Supplementary Data [file EMS137023-supplement-Supplementary_Data.zip › bc-110_roue-leGall/s2-bc-110_roue-leGall.pdf]

Legend timeline

CRAFT-related event

CUSSH Workshop

CUSSH meeting
involving local
stakeholders

Scope of work

Iterative exchanges
between academics &
Rennes city stakeholders

Stakeholders taking part to the event

2017

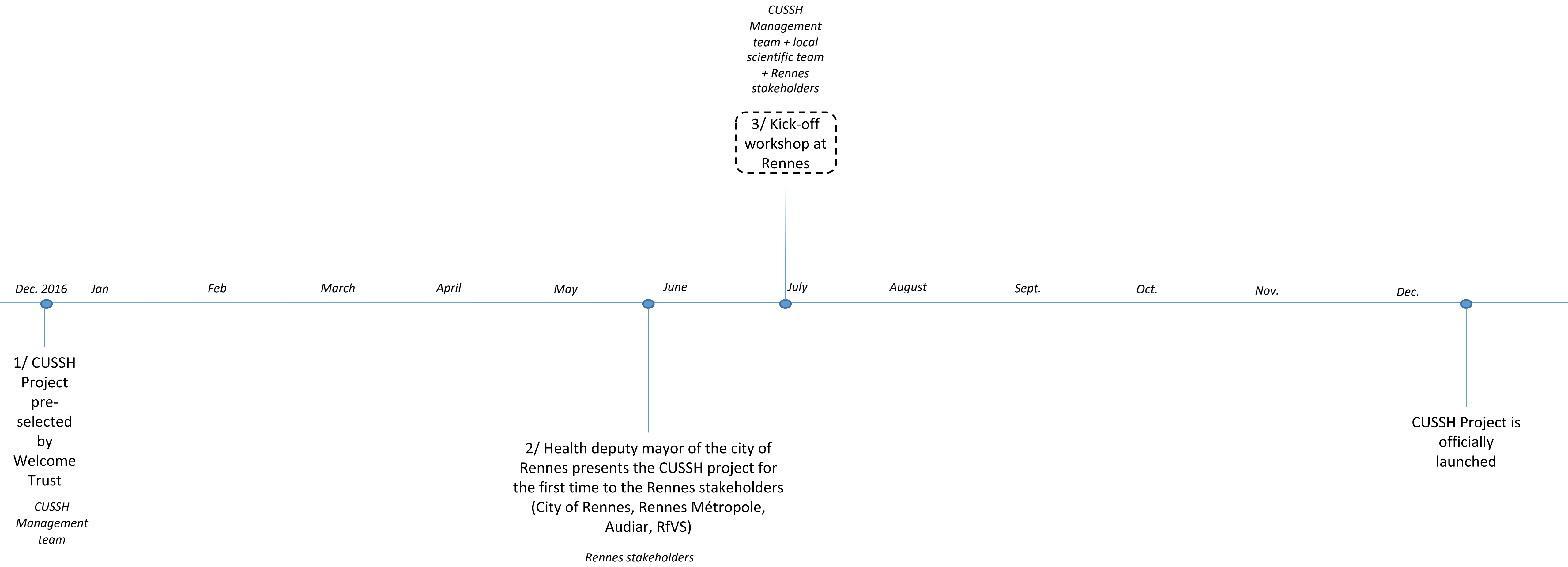
CUSSH Management team + local scientific team + Rennes stakeholders
3/ Kick-off workshop at Rennes
Dec. 2016
Jan
Feb
March
April
May
June
July
August
Sept.
Oct.
Nov.
Dec.
1/ CUSSH Project pre-selected by Welcome Trust
CUSSH Management team
2/ Health deputy mayor of the city of Rennes presents the CUSSH project for the first time to the Rennes stakeholders (City of Rennes, Rennes Métropole, Audiar, RfVS)
Rennes stakeholders
CUSSH Project is officially launched

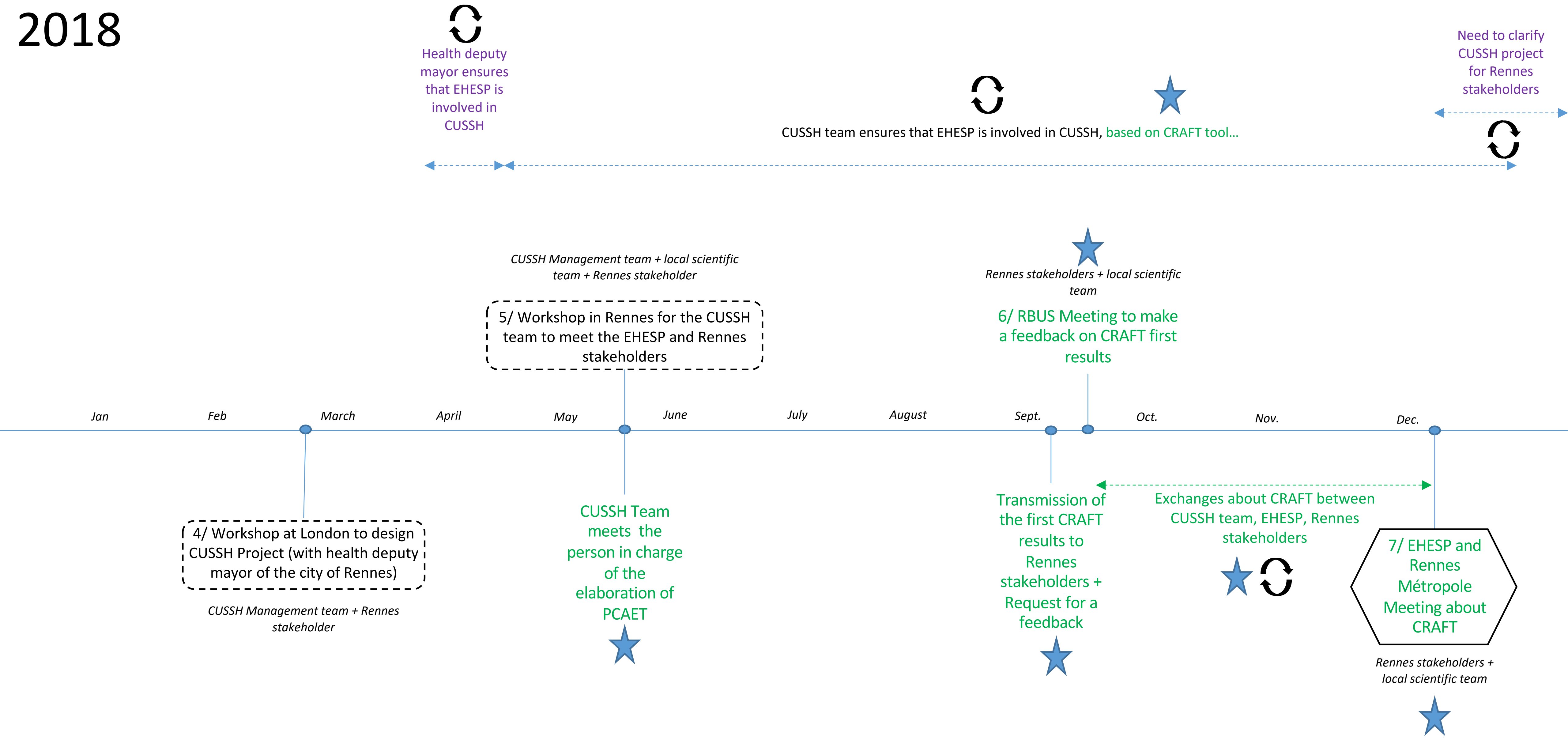
2018
Health deputy mayor ensures that EHESP is involved in CUSSH
CUSSH team ensures that EHESP is involved in CUSSH, based on CRAFT tool…
Need to clarify CUSSH project for Rennes stakeholders
CUSSH Management team + local scientific team + Rennes stakeholder
5/ Workshop in Rennes for the CUSSH team to meet the EHESP and Rennes stakeholders
Rennes stakeholders + local scientific team
6/ RBUS Meeting to make a feedback on CRAFT first results
Jan
Feb
March
April
May
June
July
August
Sept.
Oct.
Nov.
Dec.
4/ Workshop at London to design CUSSH Project (with health deputy mayor of the city of Rennes)
CUSSH Management team + Rennes stakeholder
CUSSH Team meets the person in charge of the elaboration of PCAET
Transmission of the first CRAFT results to Rennes stakeholders + Request for a feedback
Exchanges about CRAFT between CUSSH team, EHESP, Rennes stakeholders
7/ EHESP and Rennes Métropole Meeting about CRAFT
Rennes stakeholders + local scientific team

2019

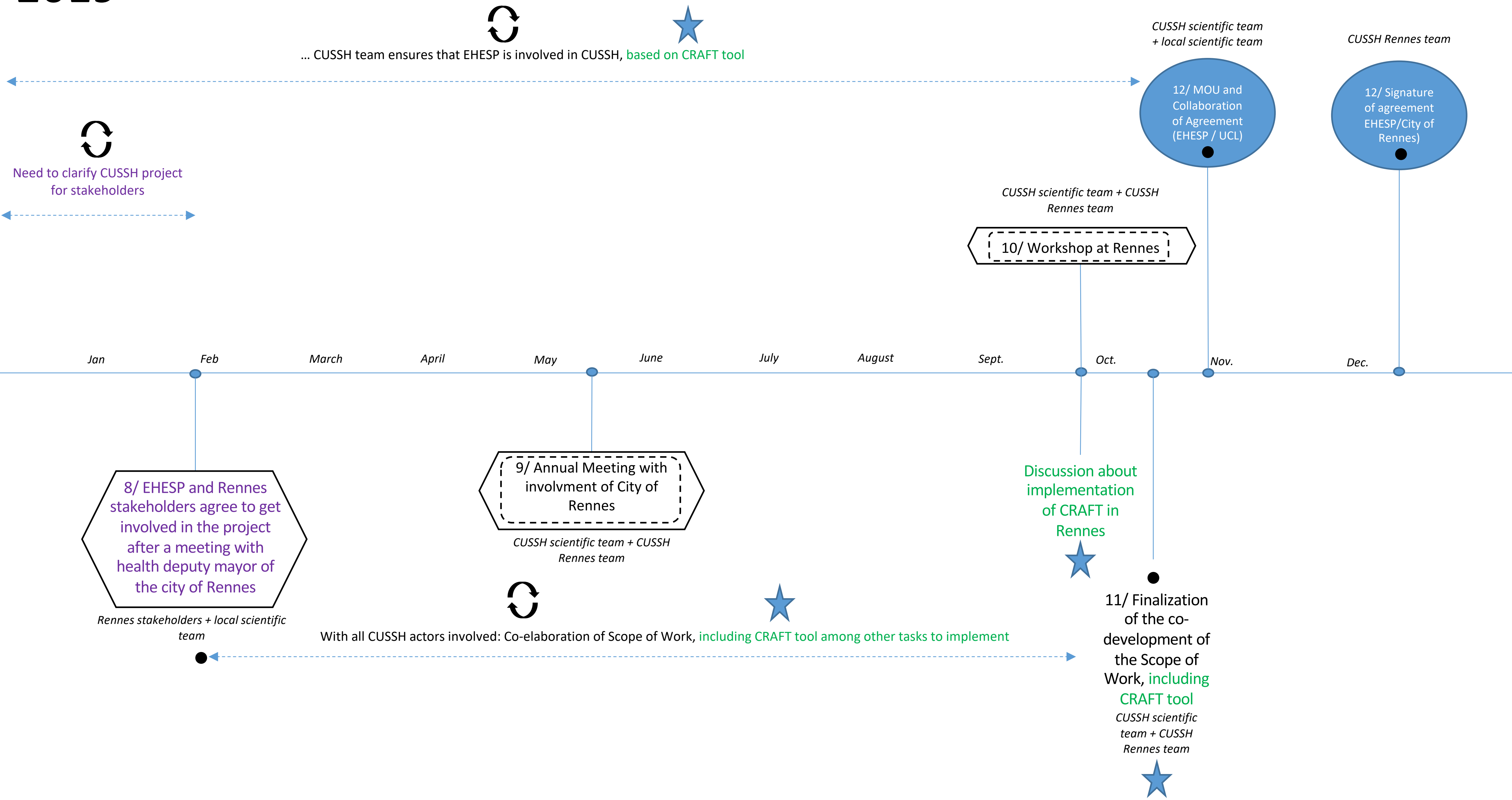

... CUSSH team ensures that EHESP is involved in CUSSH, based on CRAFT tool
Need to clarify CUSSH project for stakeholders
CUSSH scientific team + local scientific team
12/ MOU and Collaboration of Agreement (EHESP / UCL)
CUSSH Rennes team
12/ Signature of agreement EHESP/City of Rennes)
CUSSH scientific team + CUSSH Rennes team
10/ Workshop at Rennes
Jan
Feb
March
April
May
June
July
August
Sept.
Oct.
Nov.
Dec.
8/ EHESP and Rennes stakeholders agree to get involved in the project after a meeting with health deputy mayor of the city of Rennes
Rennes stakeholders + local scientific team
9/ Annual Meeting with involvment of City of Rennes
CUSSH scientific team + CUSSH Rennes team
Discussion about implementation of CRAFT in Rennes
With all CUSSH actors involved: Co-elaboration of Scope of Work, including CRAFT tool among other tasks to implement
11/ Finalization of the co-development of the Scope of Work, including CRAFT tool
CUSSH scientific team + CUSSH Rennes team

2020

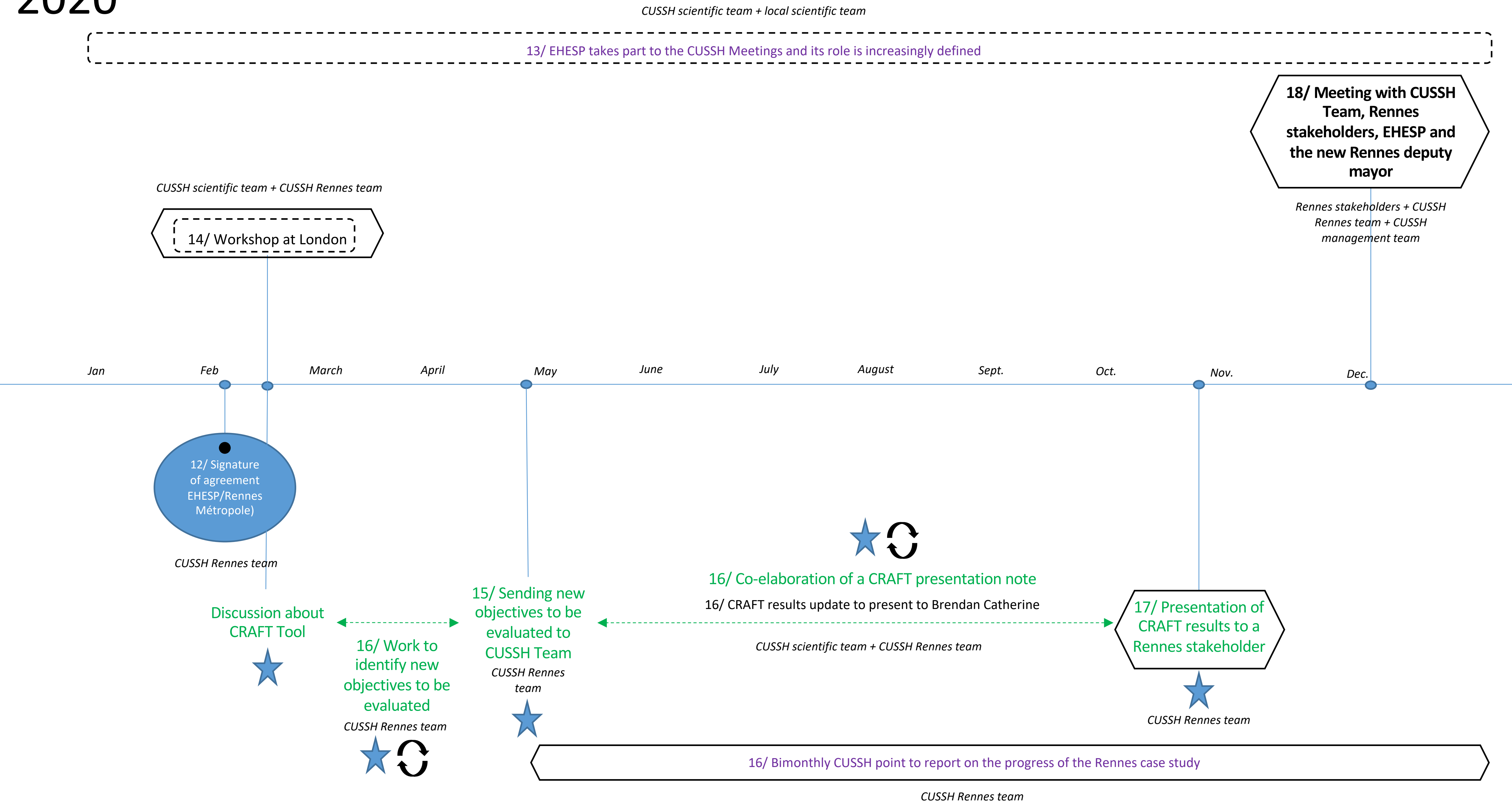

CUSSH scientific team + local scientific team
13/ EHESP takes part to the CUSSH Meetings and its role is increasingly defined
18/ Meeting with CUSSH Team, Rennes stakeholders, EHESP and the new Rennes deputy mayor
Rennes stakeholders + CUSSH Rennes team + CUSSH management team
CUSSH scientific team + CUSSH Rennes team
14/ Workshop at London
Jan
Feb
March
April
May
June
July
August
Sept.
Oct.
Nov.
Dec.
12/ Signature of agreement EHESP/Rennes Métropole)
CUSSH Rennes team
Discussion about CRAFT Tool
16/ Work to identify new objectives to be evaluated
CUSSH Rennes team
15/ Sending new objectives to be evaluated to CUSSH Team
CUSSH Rennes team
16/ Co-elaboration of a CRAFT presentation note
16/ CRAFT results update to present to Brendan Catherine
CUSSH scientific team + CUSSH Rennes team
17/ Presentation of CRAFT results to a Rennes stakeholder
CUSSH Rennes team
16/ Bimonthly CUSSH point to report on the progress of the Rennes case study
CUSSH Rennes team
